# Urine Drug Testing at an Ambulatory Pain Clinic in Patients Receiving Opioids for Cancer Pain †

**DOI:** 10.3390/cancers18193068

**Published:** 2026-09-22

**Authors:** Jaya Amaram-Davila, Varsha Pawate, Sriram Yennurajalingam, Ali Mehdi Rizvi, Rosheni Kandaswamy, Reaundra Mccullough-Roach, Sonal Admane, Min Ji Kim, Akhila Reddy, Lei Feng, Larry Driver, Eduardo Bruera, Joseph A. Arthur, Dhanalakshmi Koyyalagunta

**Affiliations:** 1 Department of Palliative, Rehabilitation and Integrative Medicine, The University of Texas MD Anderson Cancer Center, 1515 Holcombe Boulevard, Unit 1414, Houston, TX 77030, USA; 2 Department of Internal Medicine, University of California Davis, Sacramento, CA 95817, USA; 3 Department of Pain Medicine, The University of Texas MD Anderson Cancer Center, Houston, TX 77030, USA; 4 Department of Biostatistics, The University of Texas MD Anderson Cancer Center, Houston, TX 77030, USA

**Keywords:** opioids, cancer pain, urine drug test, opioid monitoring, safe prescription

## Abstract

**Importance:** Urine drug test (UDT) can help screen and monitor aberrant controlled substance use among patients requiring opioid therapy for cancer pain. However, data on UDT utilization in patients with cancer pain remains limited. **Objective:** To determine the rate of UDT ordering and the factors associated with UDT order placement and the frequency and type of abnormal UDT findings. **Methods:** This retrospective study included adults (≥18 yrs) with a cancer diagnosis receiving prescription opioids for pain in an ambulatory pain clinic from November 2017 through April 2018. Descriptive statistics summarized patient characteristics and UDT findings. Logistic regression was used to identify factors associated with UDT ordering. **Results:** Five hundred fifty-eight patients were included in the study; the median age was 61 years, 52% were female, 74% were White, and 93% had active cancer. One hundred forty-one patients (25%) had UDT orders. On multivariate analysis, UDT ordering was associated with SOAPP-14 score ≥ 7 (OR, 2.03 95% CI 1.19–3.47 *p* = 0.009) and a history of substance use disorder (OR, 2.87 95% CI: 1.39–5.75 *p* = 0.004). Among those tested, 49 (35%) had abnormal results. A total of 80 abnormalities were identified, with some UDTs showing multiple findings; the most common was the absence of prescribed opioids (48%). **Conclusions:** Only one in four patients receiving opioids in an ambulatory cancer pain clinic received a UDT order, suggesting a more selective rather than routine use. UDT ordering was more common among patients with a SOAPP-14 score ≥ 7 and a history of substance use disorder. Among patients who underwent UDT, approximately one-third had abnormal results, with the absence of prescribed opioids or their expected metabolites being the most common finding. These findings suggest that UDT may provide important clinically relevant information as a component of comprehensive opioid monitoring. Further research is needed to establish evidence-based approaches to UDT implementation that promote consistent opioid safety practices.

## Simple Summary

In this retrospective study of 558 patients with cancer pain receiving opioid therapy, urine drug test (UDT) was ordered in 25% of patients, suggesting selective rather than routine use. Among the 141 patients tested, 35% had abnormal UDT results, with the absence of prescribed opioids or their expected metabolites being the most common finding. These findings highlight the potential role of UDT as a component of comprehensive opioid monitoring and the need for evidence-based guidance to support consistent UDT use in cancer pain management.

## 1. Introduction

Urine drug test (UDT) is an important tool for monitoring adherence in patients receiving opioid therapy for cancer pain [1,2]. It can identify the presence of prescribed medications and detect illicit or non-prescribed controlled substances, thereby supporting safer prescribing practices [1,2,3]. In 2023, nearly 8.6 million Americans aged 12 years and older reported using unprescribed opioids, often obtained from friends or family members, in the prior 12 months [4]. Similar to the general population, patients with cancer may also be at risk for misuse of prescribed controlled substances, highlighting the need for close monitoring [2,5,6,7,8]. In a study by Rauenzahn et al., 70% of patients with cancer who underwent UDT had inappropriate results; of them, patients with risk factors for opioid misuse (46%) had an absence of prescribed opioids or the presence of an unprescribed controlled substance or illicit drugs in the UDT [9]. Despite its potential to improve safe opioid prescription at their initiation, titration, and maintenance, the lack of standardized recommendations for UDT in patients with cancer on opioid therapy leaves clinicians to decide who should be tested and when.

Like general populations, patients with cancer carry risk factors for aberrant use of controlled substances and are equally vulnerable to the risks associated with it, such as, but not limited to, overdose and death. A few studies have shown how aberrant behaviors can interfere with anti-cancer therapies and increase all-cause mortality in these patients [5,6,10]. In such cases, optimal utilization of UDT can help monitor adherence to prescribed controlled substances and detect aberrant behaviors in a timely fashion [11]. The National Comprehensive Cancer Network (NCCN) Adult Pain Guidelines state that UDT should be considered in patients receiving opioid therapy when feasible during opioid initiation, titration, and maintenance [1,12]. In addition, NCCN guidelines encourage clinicians to consider it as part of comprehensive opioid management in patients with subacute and chronic cancer pain [1,12,13]. Despite this guidance, evidence remains limited on UDT use in routine cancer pain practice, including which patients are selected for testing and what findings are identified when testing is performed. Consequently, UDT use may vary based on clinician discretion and perceived patient risk [14,15,16].

Although UDT is recognized as a component of comprehensive opioid management, its implementation in cancer pain practice remains uncertain. Current guidance allows clinicians to consider UDT based on clinical circumstances [1,13]. Still, it provides limited direction regarding who to test, when to test, how frequently to test, and how to apply those results in clinical practice, particularly in the context of patient-level risk factors [13]. Selective testing may be clinically appropriate in some circumstances; however, it may also create variability in testing practices across patients and clinical settings [2,17,18]. Moreover, the characteristics of patients who are selected for testing and the frequency and nature of unexpected findings among those tested are not well established in cancer pain populations. Understanding real-world patterns of UDT utilization may therefore help clarify how clinicians currently operationalize available guidance and identify opportunities to support more consistent, risk-informed opioid monitoring. Data describing both the factors associated with UDT ordering and the findings identified through testing are needed to inform future implementation strategies and clinical guidance.

Thus, the main objective of this study was to determine the utilization rate of UDT orders among patients receiving opioid therapy for cancer pain in an ambulatory pain management clinic at a comprehensive cancer center. We also examined the factors associated with UDT orders, the types and frequency of abnormal UDT results, and the risk factors related to these results.

## 2. Methods

### 2.1. Study Design and Population

This retrospective study was conducted to evaluate the real-world utilization of UDT among patients receiving opioid therapy for cancer pain in an ambulatory pain management clinic at the University of Texas MD Anderson Cancer Center (UTMDACC).

The study included patients seen at the UTMDACC Ambulatory Pain Management Clinic between November 2017 and April 2018. Eligible patients were adults (≥18 years) with a past or current diagnosis of cancer who received prescription opioids for cancer pain during the study period. The UTMDACC Institutional Review Board approved this retrospective study.

### 2.2. Clinic Process and Instruments

The ambulatory pain management clinic provides five full-day services at the main campus and 3 full-day services per week at each of 4 satellite locations. During the study period, the clinic was staffed by 9 board-certified physicians, 1 psychologist, 5 trainees, 5 advanced practice providers, and 4 pain medicine-trained registered nurses (RN). The clinic predominantly serves patients with advanced cancer, with a smaller proportion of patients with early-stage disease, early remission, or no evidence of disease.

As part of the clinic’s opioid management process, clinicians were encouraged to order a baseline UDT at the initial consultation. They could order repeat UDTs during subsequent follow-up visits based on clinical judgment. During the study period, each patient had only one UDT order recorded. After a UDT was ordered, patients were asked to provide a urine specimen, which was labeled and submitted to the MD Anderson laboratory for toxicology testing. In some instances, the UDT order was canceled when a patient was unable to provide a urine specimen. All patients included in the UDT group in this study provided a urine specimen and underwent toxicology testing.

The pain management clinic uses standardized approaches to symptom assessment and opioid risk assessment, including the Edmonton Symptom Assessment Scale—Financial and Spiritual Distress (ESAS-FS) [19] and opioid risk assessment instruments such as the Cut Down, Annoyed, Guilty, and Eye-opener—Adapted to Include Drugs (CAGE-AID) [14,19] and the Screener and Opioid Assessment for Patients with Pain–14 item questionnaire (SOAPP-14) [14,19]. These previously established approaches informed the clinical variables and risk assessment measures collected in the present study; however, the study was independently designed to examine UDT utilization, factors associated with UDT ordering, and the frequency and types of abnormal UDT findings in patients with cancer.

The ESAS-FS 12-item scale is a patient-administered, validated tool used to assess cancer-related symptoms—pain, fatigue, nausea, depression, anxiety, drowsiness, shortness of breath, appetite, feelings of well-being, sleep, financial distress, and spiritual pain—on a numerical scale ranging from 0 to 10 (0: no symptoms, 10: worst possible symptoms). The ESAS-FS evolved from the original 10-item ESAS by adding two components—financial distress and spiritual pain [20]. ESAS-FS questionnaires were administered during all patient visits to our clinic. They assess patients’ responses to the pain regimen and overall well-being [21,22].

CAGE-AID is a clinician-administered 4-item questionnaire. It helps identify the lifetime risk of alcohol and substance use disorder (SUD). A score of ≥2 is considered positive, indicating that the individual is at risk for alcohol or illicit drug use or carries a high potential for aberrant opioid use [14,23,24].

SOAPP-14 is a patient-administered 14-item questionnaire developed to assess factors associated with potential opioid misuse. The questionnaire evaluates several domains, including history of SUD, antisocial behavior, medication-related behaviors, patient–clinician interactions, and psychiatric and neurobiological factors. Each item is rated on a 5-point Likert scale from 0 (never) to 4 (very often), with total scores ranging from 0 to 56. In this study, a total score of ≥7 was used as the threshold for increased potential for opioid misuse behaviors. This threshold was selected based on prior validation work demonstrating high sensitivity for identifying patients at risk, while recognizing that the lower specificity at this cutoff may result in false-positive classifications [14,25,26].

### 2.3. Urine Drug Test

UDT results were considered normal if they were consistent with the patient’s reported opioid use at the time of UDT collection. Results were considered abnormal if they showed one or more of the following: (1) the presence of illicit drugs such as, but not limited to, amphetamine, cocaine, or heroin; (2) the presence of unprescribed opioids or other controlled substances; or (3) the absence of a prescribed opioid or its expected metabolite in a patient who reported taking the medication [3,27]. When interpreting negative UDT results, clinicians must consider factors that may contribute to them, including assay sensitivity, timing of opioid ingestion, intermittent opioid use, opioid metabolism, and laboratory detection thresholds. The UDT can be quantitative or qualitative [3,18]. The UDT performed for patients in this study followed the Mayo Clinic Pain Clinic Survey testing algorithm [28,29]. After specimen validity testing for adulterants, urine samples underwent both a targeted opioid screen using high-resolution liquid chromatography–tandem mass spectrometry (LC-MS/MS) and an immunoassay panel. The targeted opioid screen provides definitive identification of opioids and opioid metabolites, whereas selected non-opioid drug classes screened via immunoassay undergo confirmatory testing by means of chromatography-based methods when indicated [28,29].

### 2.4. Data Collection

Patient demographics, clinical characteristics, and controlled substance-related data were obtained from the electronic medical record. The demographic and clinical variables included age, gender, race, cancer diagnosis and status, and documented history of tobacco, alcohol, marijuana, and illicit drug use. Based on the patients’ documented opioid regimens, we calculated their total morphine equivalent daily dose (MEDD), with an MEDD ≥ 90 mg being considered high [13,30]. The data also included the scores on CAGE-AID and SOAPP-14 questionnaires during their consultation visits and ESAS-FS scores collected at clinical visits. Information regarding UDT orders and results was also collected, including UDT orders placed at initial and subsequent clinic visits and the corresponding toxicology results. Abnormal UDT findings were recorded and categorized according to the predefined study criteria.

### 2.5. Outcome Measures

The primary outcome of this study was to determine the rate of UDT ordering among patients with cancer receiving opioid therapy for cancer pain. The secondary outcomes included factors associated with UDT ordering and the frequency and type of abnormal UDT results.

### 2.6. Statistical Analysis

Patients’ demographic, treatment, and clinical outcome data were summarized using descriptive statistics. For the primary outcome, the Wilcoxon rank-sum test was used to compare continuous variables between patients with and without UDT orders. The chi-square or Fisher’s exact test was used to evaluate the association between categorical variables. Among patients who underwent UDT, characteristics were compared between patients with normal and abnormal UDT results. For descriptive analyses, denominators were based on the number of patients with available data for each variable. We used a multivariable logistic regression model to identify factors independently associated with UDT ordering. Covariates included age (dichotomized as <60 vs. ≥60 years), history of SUD, SOAPP-14 score (dichotomized as ≥7 vs. <7), MEDD, and ESAS-FS total distress score on the day of UDT order placement. These covariates were selected based on their clinical relevance to opioid-related risk, opioid exposure, and symptom burden, as well as the prior literature informing opioid risk assessment in patients with cancer [14,27]. Patients with missing values for any covariate were excluded from the respective analysis (complete-case analysis). No imputation was performed. A *p*-value of <0.05 was considered statistically significant. SAS 9.4 (SAS, Cary, NC, USA) and S-Plus 8.2 (TIBCO Software Inc., Palo Alto, CA, USA) were used for all statistical analyses.

This study followed the Strengthening the Reporting of Observational Studies in Epidemiology (STROBE) guidelines. This manuscript was written, edited, and revised in accordance with STROBE guidelines [31,31].

## 3. Results

### 3.1. Patient Characteristics

Table 1 summarizes the demographics and clinical characteristics of the 558 patients seen at the clinic. The median age was 61 years (range, 18–93 years); of the patients, 288 (52%) were female, 409 (74%) were White, and 500 (93%) had active cancer. One hundred and seventy-one patients (32%) had a documented history of alcohol use, and two hundred and ninety-two (54%) had a current or previous history of tobacco smoking. The baseline median MEDD was 34 mg (Interquartile Range 15.0, 70.0).

### 3.2. Frequency of UDT Orders and Factors Associated with UDT Order Placement

Twenty-five percent of the patients (141/558) had a UDT order. UDT orders were more common among patients with one or more of the following risk factors SOAPP-14 score ≥ 7 (*p* < 0.0001), a history of SUD (*p* < 0.0001), a history of tobacco smoking (*p* = 0.006), a higher MEDD (*p* < 0.0001), younger age (median 57 y vs. 61 y; *p* = 0.002), higher ESAS-FS depression (*p* = 0.002), higher ESAS-FS anxiety (*p* = 0.002), and higher ESAS-FS total distress scores (*p* = 0.0001).

Table 2 shows a multivariable regression analysis. Of the 558 patients, 448 had complete data for the variables included in the analysis. Age < 60 years old (odds ratio [OR], 1.57; 95% CI, 0.99–2.48; *p* = 0.05); a history of SUD (OR, 2.87; 95% CI 1.39–5.75; *p* = 0.004); SOAPP-14 score ≥ 7 (OR, 2.03; 95% (CI 1.19–3.47; *p* = *0*.009); MEDD at the time of UDT ordering (OR, 1.004; 95% CI 1.001–1.008; *p* = 0.02); and ESAS-FS total distress score (OR, 1.011; 95% CI 0.999–1.022; *p* = 0.07) were independently associated with UDT order placement.

### 3.3. UDT Results and Factors Associated with Abnormal Results

Table 3 contains data on the 141 patients who underwent UDT. Of the 141 patients, 49 (35%) had abnormal UDT results; this rate is limited to the abnormal findings among tested patients only. The rate of abnormal UDT was significantly higher among those with SOAPP-14 score ≥ 7 (*p* = 0.04) and those with a history of smoking (*p* = 0.06).

### 3.4. Abnormalities in UDT Results

We identified 80 abnormalities among the 49 abnormal UDT results, as shown in Figure 1. Of the 80 UDT abnormalities, 38 (48%) were due to the absence of the prescribed opioid in the urine, with hydrocodone (37%) and fentanyl (21%) being the most common. Fourteen (18%) abnormalities were from the presence of unprescribed controlled substances, with benzodiazepines (50%) being the most common, and twenty-eight (35%) abnormalities were from the presence of illicit drugs, of which marijuana or Tetrahydrocannabinol (THC) were the most common (71%). Some patients had more than one abnormality. Percentages represent the distribution of the 80 abnormal findings and not the proportion of patients.

## 4. Discussion

The primary finding of our study is that only 25% of patients with cancer receiving opioid therapy for cancer pain at our ambulatory pain management clinic had a UDT order during the study period. This finding is consistent with previous studies demonstrating relatively low or variable UDT utilization among clinicians caring for patients with cancer pain and those receiving opioid therapy [2,9,17,32,33]. Although UDT is one component of opioid safety monitoring, its use may vary substantially across clinical settings [9,17,34]. Without standardized institutional criteria, clinicians may rely on individual clinical judgment when deciding whether to order a urine drug test. Our findings therefore highlight an opportunity to examine whether more consistent approaches to UDT ordering could strengthen opioid safety practices in cancer pain management.

Our findings suggest that clinicians used UDT selectively, and patients with recognized risk factors were more likely to receive a UDT order. A SOAPP-14 score ≥ 7, a history of SUD, or higher MEDD were independently associated with UDT ordering [35]. These findings are consistent with the previous literature identifying younger age, SUD, and higher opioid use marks as risk factors associated with aberrant opioid-related behaviors. The observed pattern may therefore reflect appropriate clinical risk assessment, with clinicians directing UDT toward patients perceived as higher risk [3,14,36]. However, reliance solely on recognized risk factors may leave some patients with potentially concerning medication-related behaviors unidentified [11,27]. Patients without an obvious history of SUD or a positive risk assessment may still have unrecognized substance use or medication-related concerns, and risk may evolve during the course of cancer treatment [2].

Among the patients who underwent UDT, 35% had an abnormal result, consistent with previous studies in both cancer and noncancer patients receiving opioid therapy [27,37]. In a previous study, Arthur et al. found that approximately 1 in 4 patients receiving opioid therapy for cancer pain had abnormal UDT findings; younger age and a positive CAGE-AID score were associated with abnormal results [27]. Similarly, in a study of 798 adults receiving opioid therapy for noncancer pain, active tobacco use was strongly associated with opioid use disorder (OR, 14; 95% CI, 9.5–20.6; *p* < 0.001) [37]. In our study, abnormal results were more common among those with a current or previous smoking history and those with a SOAPP-14 score ≥ 7, highlighting these factors as potential markers for increased opioid-related risk. Because UDT was ordered selectively in our study, the 35% rate represents the proportion of abnormal results among patients who underwent testing and should not be interpreted as the prevalence of abnormal UDT findings in the entire cohort. It is crucial to note that clinicians should exercise caution when interpreting abnormal UDT results and consider the findings in the context of the overall clinical picture. The timing of the patient’s last opioid dose, medication-taking patterns, and the possibility of false-positive or false-negative results should be considered before drawing conclusions. Discussing unexpected results with patients also provides an opportunity to clarify medication use, address potential concerns, and reinforce education regarding safe opioid use [38,39,40].

The most common abnormal UDT finding in our study was the absence of a prescribed opioid, with hydrocodone and fentanyl among the most frequently absent medications. An unexpected absence of a prescribed opioid can have several possible explanations, including inconsistent medication use, self-discontinuation, medication sharing, diversion, timing of the last dose, metabolism, or limitations related to the assay or detection threshold [4,39,41,42]. Therefore, an unexpected negative result should not automatically be interpreted as evidence of diversion or other aberrant behavior. Instead, an unexpected result should prompt further clinical assessment. Other abnormalities in UDT results included the presence of illicit substances and unprescribed controlled substances, including THC, benzodiazepines, and non-prescribed opioids. Clinicians must carefully review the timing of the patient’s last dose, medication-taking patterns, other prescribed medications, and the circumstances surrounding the result. When appropriate, additional testing or consultation with the laboratory may help clarify these findings. This approach is particularly important in patients with cancer, in whom changes in opioid requirements, treatment-related symptoms, adverse effects, and medication self-adjustment may occur frequently [40].

In our study, THC/marijuana was the most detected illicit substance among abnormal UDT findings. This classification should be interpreted carefully in the context of the evolving legal landscape: although cannabis use is legal in some U.S. states, Texas permitted only limited medical cannabis use through the Compassionate Use Program during the study period [43]. In addition, over-the-counter cannabidiol (CBD) products may contain trace amounts of THC that can be detected on urine toxicology [18,44]. Given the perception that THC- and CBD-containing products may help alleviate cancer-related symptoms, their use among patients with cancer is not uncommon [45,46]. A positive THC result therefore provides an opportunity for clinicians to discuss cannabinoid use with patients and counsel them regarding potential interactions, safety considerations, and opioid-related risks.

Our findings highlight the potential role of UDT as one component of comprehensive opioid monitoring in cancer pain management. Standardized institutional criteria for UDT ordering may promote more consistent opioid safety practices while allowing clinicians to individualize monitoring based on patient circumstances by combining them with thorough history, medication review, review of PDMP monitoring, and use of opioid risk assessment tools (SOAPP-14 and CAGE-AD, etc.). This study also provides insight into real-world opioid monitoring practices in a specialized ambulatory pain management clinic and identifies opportunities to strengthen opioid safety in cancer pain management.

Several limitations should be considered. This retrospective, single-center study was conducted at a specialized clinic within a tertiary cancer center, which may limit generalizability. UDT ordering was determined by individual clinicians based on perceived clinical risk, introducing potential selection bias and confounding by indication. Therefore, the 35% rate of abnormal UDT findings among tested patients should not be interpreted as the prevalence of abnormal UDT results in the entire cohort, and more frequent or consistent testing might have identified additional findings. UDT results may also be influenced by medication timing, metabolism, assay characteristics, and detection thresholds and therefore require clinical interpretation rather than being considered definitive evidence of aberrant opioid use. Finally, the study period preceded publication of the 2022 Centers for Disease Control and Prevention Clinical Practice Guideline for Prescribing Opioids for Pain [13]; thus, the findings reflect opioid prescribing and UDT practices during 2017–2018 and may not fully represent current clinical practice.

## 5. Conclusions

In this retrospective study of patients receiving opioid therapy for cancer pain in a specialized ambulatory pain management clinic, UDT was performed in approximately one in four patients, suggesting that testing was used selectively rather than routinely, especially among those with pre-existing risks for aberrant opioid use such as a SOAPP-14 score ≥ 7, a history of SUD, or higher MEDD. Among patients who underwent testing, more than one-third had abnormal UDT findings, most commonly the absence of prescribed opioids or their expected metabolites. Current or previous tobacco use and a SOAPP-14 score ≥ 7 were associated with abnormal UDT findings, suggesting that clinical risk factors may influence the likelihood of identifying unexpected results. However, UDT findings require careful interpretation in the context of medication use, timing of the last opioid dose, and other clinical factors and should be discussed with patients in a nonjudgmental manner. These findings support the potential role of UDT as one component of comprehensive opioid monitoring and highlight the need for evidence-based, standardized guidance to support consistent and individualized UDT practices and opioid stewardship in patients with cancer.

## Figures and Tables

**Figure 1:**
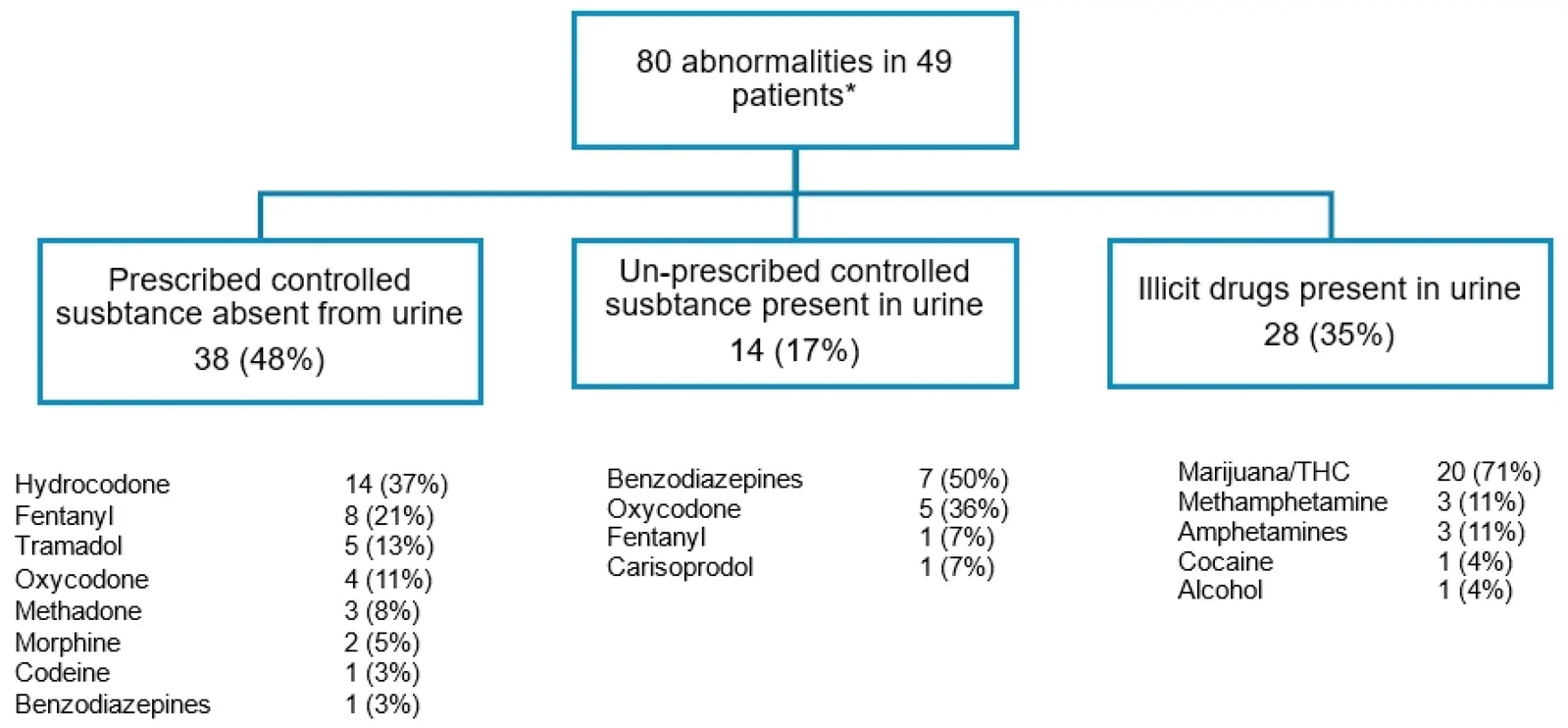
Frequency of abnormal urine test results and types of abnormalities. Percentage distribution represents the 80 abnormalities among the 49 abnormal UDT results and are not patient-level proportions. * Some patients have more than 1 urine abnormality.

**Table 1:** Patient Characteristics Based on Urine Drug Testing Order Status.

No. of Patients (%) *				
Characteristic	All Patients N = 558 (%)	NO UDT *n* = 417 (75%)	UDT *n* = 141 (25%)	*p*
Age: Median [range], y	61 [18–93]	61 [19–93]	57 [18–90]	0.002 ^a^
Female	288 (52)	216 (52)	72 (52)	0.88
**Race**				
White	409/553 (74)	304/413 (74)	105/140 (75)	0.74
Black	76/553 (14)	55/413 (13)	21/140 (15)	
Asian	18/553 (3)	16/413 (4)	2/140 (1)	
Other	50/553 (9)	38/413 (9)	12/140 (9)	
Missing	5	4	1	
**Cancer Status**				
NED	37/537 (7)	27/403 (7)	10/134 (7)	0.76
Active cancer	500/537 (93)	376/403 (93)	124/134 (93)	
Missing	21	14	7	
**Opioid Risk Assessment screening tools:**				
SOAPP-14 ≥ 7	97/477 (20)	54/350 (15)	43/127 (34)	<0.0001 ^a^
CAGE-AID ≥ 2	18/468 (4)	8/344 (2)	10/124 (8)	0.004 ^a^
History of substance use disorder (Yes)	47/528 (9)	20/395 (5)	27/133 (20)	<0.0001 ^a^
Smoking History				0.006 ^a^
Never	249/541 (46)	196/403 (49)	53/138 (38)	
Former	207/541 (38)	155/403 (39)	52/138 (38)	
Current	85/541 (16)	52/403 (13)	33/138 (24)	
Missing	17	14	3	
Alcohol History (Yes)	171/533 (32)	128/398 (32)	43/135 (32)	0.95
**ESAS-FS, Median [IQR]**				
Pain	7 [5.0, 9.0]	7 [5.0, 8.0]	8 [5.0, 9.0]	0.03 ^a^
Fatigue	6 [4.0, 8.0]	6 [3.0, 8.0]	6 [4.0, 8.0]	0.09
Nausea	0 [0.0, 3.0]	0 [0.0, 3.0]	0 [0.0, 4.0]	0.24
Depression	0 [0.0, 4.0]	0 [0.0, 4.0]	2 [0.0, 6.0]	0.002 ^a^
Anxiety	2 [0.0, 5.0]	2 [0.0, 5.0]	4 [0.0, 7.0]	0.002 ^a^
Drowsiness	3 [0.0, 5.0]	3 [0.0, 5.0]	3 [0.0, 6.0]	0.53
Shortness of breath	0 [0.0, 3.0]	0 [0.0, 3.0]	0 [0.0, 5.0]	0.16
Appetite	4 [1.0, 6.0]	4 [1.0, 6.0]	5 [1.0, 6.0]	0.39
Emotional well-being	5 [2.0, 6.0]	4 [2.0, 6.0]	5 [2.0, 7.0]	0.03 ^a^
Sleep	5 [3.0, 8.0]	5 [3.0, 7.0]	6 [3.0, 8.0]	0.06
Financial Distress	1 [0.0, 5.0]	0 [0.0, 4.0]	3 [0.0, 6.0]	0.0001 ^a^
Spiritual Pain	0 [0.0, 2.0]	0 [0.0, 1.0]	0 [0.0, 3.0]	0.04 ^a^
ESAS-FS Total Distress Score	39 [26.0, 53.0]	38 [25.0, 49.0]	43 [30.0, 57.0]	0.002 ^a^
MEDD, Median [IQR], mg	34 [15.0, 70.0]	30 [10.0, 60.0]	45 [20.0, 90.0]	<0.0001 ^a^

* Some characteristics have missing information. ^a^ *p* < 0.05 was considered statistically significant. Abbreviations: UDT: Urine drug testing; NED: No evidence of disease; SOAPP-14: Screener and Opioid Assessment for Patients with Pain-14 items; CAGE-AID: Cut down, Annoyed, Guilty, and Eye-opener—Adapted to Include Drug Use. ESAS-FS: Edmonton Symptom Assessment System—Financial and Spiritual Distress; IQR: Interquartile Range; MEDD: Morphine Equivalent Daily Dose.

**Table 2:** Multivariable Logistic Regression Model for Predictors of Urine Drug Test Ordering.

Covariate	Effect	OR ^e^	95% CI ^f^	*p*
Age ^a^	<60 y vs. ≥60 y	1.569	0.994–2.479	0.05
History of substance use disorder	Yes vs. No	2.837	1.399–5.753	0.004
Total SOAPP-14 ^a,b^ Score	≥7 vs. <7	2.032	1.191–3.467	0.009
MEDD ^c^		1.004	1.001–1.008	0.02
ESAS-FS ^d^ Total Distress Score		1.011	0.999–1.022	0.07

^a^ Age and SOAPP-14 were analyzed as the dichotomized variables. Abbreviations: ^b^ SOAPP-14, Screener and Opioid Assessment for Patients with Pain—14 items; ^c^ MEDD, morphine equivalent daily dose; ^d^ ESAS-FS, Edmonton Symptom Assessment System—Financial and Spiritual Distress; MEDD, morphine equivalent daily dose; ^e^ OR: Odds Ratio; ^f^ CI, confidence interval. Note: Multivariable logistic regression was performed among 448 patients with complete data for all covariates included in the model. The outcome was UDT order placement.

**Table 3:** Characteristics of patients with abnormal and normal urine drug test results (N = 141).

Characteristic	No. (%) of Patients ^a^		*p* ^b^
	Abnormal UDT ^c^ *n* = 49	Normal UDT ^c^ *n* = 92	
Age: <60	35 (71)	49 (53)	0.04
Male	26 (53)	43 (47)	0.48
**Race**			
White	37 (77)	68 (74)	0.83
Black	6 (13)	15 (16)	
Others	5 (10)	9 (10)	
**Cancer Status**			
Active Cancer	43 (92)	81 (93)	0.74
NED ^d^	4 (9)	6 (7)	
History of substance use disorder (YES)	12 (26)	15 (17)	0.26
**Smoking History**			
Never	14 (30)	39 (43)	0.06
Former	17 (35)	35 (39)	
Current	17 (35)	16 (18)	
Alcohol History (YES)	16 (35)	27 (30)	0.69
SOAPP-14 ^e^ ≥ 7	20 (47)	23 (27)	0.04
CAGE-AID ^f^ ≥ 2	6 (14)	4 (5)	0.09

^a^ Some characteristics have missing information. ^b^ *p* < 0.05 was considered significant. Abbreviations: ^c^ UDT: urine drug testing; ^d^ NED: no evidence of disease; ^e^ SOAPP-14: Screener and Opioid Assessment for Patients with Pain—14 items; ^f^ CAGE-AID: Cut down, Annoyed, Guilty, and Eye-opener—Adapted to Include Drug Use.

## Data Availability

The datasets generated and/or analyzed during the current study are not publicly available due to institutional policies and patient privacy regulations but may be available from the corresponding author upon reasonable request and approval from the appropriate institutional review bodies.
